# From inequalities to vulnerability paradoxes: juxtaposing older adults’ heat mortality risk and heat experiences

**DOI:** 10.1186/s12940-025-01179-2

**Published:** 2025-04-26

**Authors:** Małgorzata Wrotek, Iulia Marginean, Zofia Boni, Franciszek Chwałczyk, Ana M. Vicedo-Cabrera, Coral Salvador, Barbara Jancewicz

**Affiliations:** 1 Centre of Migration Research, University of Warsaw, Warsaw, Poland; 2 Faculty of Economic Sciences, University of Warsaw, Warsaw, Poland; 3 CICERO Center for International Climate Research, Oslo, Norway; 4 Department of Geosciences, Meteorology and Oceanography Section, University of Oslo, Oslo, Norway; 5 Institute of Anthropology and Ethnology, Adam Mickiewicz University, Poznań, Poland; 6 Institute of Cultural Studies, Faculty of Historical and Pedagogical Sciences, University of Wrocław, Wrocław, Poland; 7 Institute of Social and Preventive Medicine, University of Bern, Bern, Switzerland; 8 Oeschger Center for Climate Change Research, University of Bern, Bern, Switzerland; 9 Centro de Investigación Mariña, Environmental Physics Laboratory (EphysLab), Universidade de Vigo, Ourense, Spain

**Keywords:** Vulnerability, Paradox, Inequalities, Climate change, Heat waves, Heat mortality, Heat experience, Heat stress, Heat risk, Older adults

## Abstract

**Background:**

Increasing temperatures across the globe, including in Europe, pose one of the biggest threats to human health and wellbeing. Different kinds of inequalities, determined by age, sex/gender, isolation, socio-economic status, occupation, living in the city, and health situation, create vulnerability factors influencing people’s heat-related mortality risk and their daily experiences during summer.

**Methods:**

Our study uses an interdisciplinary approach to research how intersecting inequalities generate vulnerabilities to heat stress among older adults (65+) in two European cities: Warsaw and Madrid. We combine three methodological approaches juxtaposing quantitative and qualitative data: (1) epidemiological analysis that uses daily mortality data in Warsaw and Madrid coupled with meteorological station temperature data from HadISD; (2) the OLS regression based on the survey conducted in Warsaw and Madrid in 2022; and (3) the focus group interviews conducted in Warsaw in 2021.

**Results:**

Our data confirms that good health and financial situation protect people both from mortality risk and negative heat experiences. Interestingly, both air conditioning (A/C) usage and being physically active increase the negative heat experiences people reported. Finally, we identified two *vulnerability paradoxes* understood as situations when a person or a group might be more at risk but not experience or perceive negative impacts of heat. These paradoxes affect the oldest adults (80+) and older people living alone in both cities.

**Conclusions:**

Studies on vulnerability and adaptation need to incorporate both large scale top-down data sets and bottom-up, localized data based on individual experience. Combining various methods and disciplinary approaches enables identification of inequality factors and vulnerability paradoxes that remain unnoticed or underestimated while increasing people’s vulnerability to heat stress.

**Supplementary Information:**

The online version contains supplementary material available at 10.1186/s12940-025-01179-2.

## Introduction

Europe, as the fastest warming continent [1], is particularly affected by increasing temperatures. Only in the summer of 2022, over 60 000 excess deaths across Europe were attributed to heat exposure and these deaths proved unequally distributed between sexes and age groups [2]. Existing research identified various inequality factors that increase heat-related risks: old age, being a woman, being of minority ethnicity, having lower socio-economic status or preexisting health conditions [3–6]. Another such population group is urban dwellers, who face higher exposure to heat stress, due to the Urban Heat Island effect [7], making cities increasingly dangerous places to live. These different intersecting inequalities generate vulnerabilities to heat stress. However, some groups that are objectively at higher risk might not experience or notice the heat’s impact on them. Without perceiving heat stress, it is unlikely that they will take precautions, which adds to their already high risk. For the remaining part of this paper, we refer to such instances as *vulnerability paradoxes*.

Inequalities are often the drivers of risk and vulnerability and refer not only to socio-economic indicators such as income or educational level, but also to sociodemographic factors such as age, sex/gender or living arrangements [8]. Such, multi-dimensional inequalities related to geographic location, economic and socio-cultural aspects cause differences in vulnerability and exposure [9].

Epidemiological analysis is useful to assess the impacts and risks of heat stress and identify the most vulnerable population groups, but its scope is limited to the most severe impacts of heat on health, based on mortality and hospitalizations data. In other words, epidemiology assesses the most devastating consequences of heat only after severe health impacts have occurred. Ensor et al. [10] argue for the need to reframe our approach to study vulnerability towards a more bottom-up perspective, accounting for interacting socio-environmental processes and individual experiences [11, 12]. This can be achieved by taking alternative epistemological starting points to research vulnerability, which allow researchers to “see” beyond the hazard-impact relationship and “look” at vulnerability and adaptation through the lens of socio-cultural transformation and local, individualized context [13]. We therefore conceptualize vulnerability not only as an outcome (mortality or morbidity) but as a process that precedes and might lead to such an outcome, focusing on inequalities that shape people’s individual perspectives (subjective perception and experience).

Our study focused on one of the vulnerable populations: people at the age of 65 years old or older living in two European cities, Madrid and Warsaw. We selected those two case study locations due to their different climates. According to the Köppen-Geiger climate classification [14], Warsaw is characterised by a humid continental climate with warm summers (Dfb), with average summer temperatures around 18–22 °C (IMiGW) while hot-summers Mediterranean climate (Csa) is representative for Madrid, with mean summer temperatures of 24–28 °C (AEMET). This difference in climate makes it valuable to look at how the inhabitants of each city experience, react and are vulnerable to heat stress.

We combine three research methods: (1) epidemiological assessments of mortality risk associated with hot temperatures for Warsaw and Madrid with (2) survey results on older adults’ perception of heat in Warsaw and Madrid, and (3) qualitative data from focus groups in Warsaw on older adults’ experiences of heat. In other words, we juxtapose the ‘objective’ risks of heat-related mortality determined through epidemiological analysis at the city level with the ‘subjective’ older adults’ perception and experiences of heat at the individual level as they result from the survey and focus group analysis, to better understand the complex processes that shape older adults’ vulnerabilities to heat. We showcase three groups of factors that point to inequalities in how older adults are affected by and experience heat: (1) demographic– especially sex/gender and age; (2) socioeconomic; and (3) health status & wellbeing. *Vulnerability paradoxes* emerge from this approach, demonstrating that the oldest group of adults (80+), and people living alone, in both cities do not notice the negative effects of heat on their bodies and wellbeing, while being at higher risk to heat stress, which might exacerbate their vulnerability. This article highlights how interdisciplinary research and the integration of top-down and bottom-up approaches can reveal people’s hidden vulnerabilities to heat stress.

## Background

Environmental epidemiology is one of the main disciplines studying how heat impacts people’s health. Administrative data on deaths and hospital admissions is commonly used in epidemiological analysis to assess the relationship between temperature, mortality [5, 15–17] and morbidity [18, 19]. Although epidemiological analysis is mostly concerned with heat exposure, an increasing number of studies have, more recently, started to assess vulnerability as well, evaluating the effect modification of demographic, socioeconomic or urban characteristics on health outcomes [19–22]. A systematic review of effect modification, identified 207 such studies published between 1980 and 2017 [6]. Our literature review includes epidemiological studies, alongside research from other disciplines that focus on individual experiences of heat stress.

### Demographic factors

Older age is the factor most shown to increase heat stress vulnerability [4, 5, 23–25]. Some studies find the age dependency to be weaker, yet still present, as in a recent analysis by Scovronick et al. [26]. Son et al. [6] find age as the most consistent effect modifier for heat-related excess mortality while Ballester et al. [2] confirmed the increased vulnerability of oldest adults (80+) to heat stress during 2022 summer in Europe. Studies also show that older adults might miss the immediate signs of strain during heat [27] and thus, forgo taking protective measures [28]. Simultaneously, older adults (60+) can experience and report more long-term heat impacts on their health [29]. Thus, evidence on mortality risk increasing with age is very strong, while for heat experiences the results are nuanced.

Many studies suggest that women have a higher risk of heat-related mortality than men [5, 15, 25, 30, 31]. However, Son’s et al. [6] systematic review found strong evidence of higher heat stress mortality risk for men in 12 and for women in 37 studies. Women’s increased mortality risk is partially driven by their greater representation in the older age cohorts, which are disproportionally susceptible to heat stress [2]. Another driver might be heat’s ability to exacerbate preexisting economic and social inequalities between the sexes [32]. Gender differences are highlighted also in studies on thermal comfort [33, 34] where women more often declare discomfort and heat-related symptoms [35, 36]. Overall, women seem to be more sensitive to heat and are often, though not always, at higher risk.

Isolation also seems to be a factor impacting heat risk, which can be exacerbated by “stay at home during heatwave” advice [37]. In his analysis of the 1995 Chicago heatwave, Klinenberg [38] identified isolation as a crucial risk, since people living alone might have no one to notice potential problems or to call an ambulance when one is needed. Several studies confirm that living alone results in a higher risk of heat-related mortality [25, 39–41]. Other studies show that a more general notion of social isolation corresponds with poorer health and higher mortality risk [41]. Thus, household size and social relationships might affect people’s heat experiences and mortality risk.

### Socioeconomic factors

Most studies emphasize the importance of socioeconomic factors for heat-related mortality or morbidity [4, 5, 19, 41]. However, the definition of socioeconomic status (SES) varies with typical indicators including education and income, measured differently and at various aggregation levels. e.g., individual/household income, self-assessed income, regional/country GDP or small area socioeconomic indices based on census information. The variability in measurements makes comparisons difficult, still, many studies do confirm that a low SES increases heat-related death risk [24, 42, 43] as well as the risk of acute cardiovascular disease [19]. People with lower SES have smaller heat adaptation capacity e.g., limited access to A/C. Furthermore, those in a difficult socio-economic situation also tend to live in districts where the Urban Heat Island effect hits strongest e.g., people of colour in the USA [3]; or poorer, less educated, more often single-person households in Madrid [44].

Looking only at one aspect of SES: education, Son’s et al. [6] systematic review found limited evidence of the importance of education since little mortality data is accompanied by education information. However, studies in Spain [45] and France [46] find that higher education is key factor in protecting from the negative heat effects. The financial situation was found to have an impact on heat risk [6, 18, 41]. In general, heatwaves are less likely to harm higher-income populations [47]. Thus, while there is strong evidence of a protective role of higher SES, there is a lack of studies how individual-level SES relates to heat risks [23].

A/C usage might be one of the links between SES and heat stress risk. Since people spend most of their time indoors [48], indoor temperatures are a crucial heat-related risk factor [49]. Aside from building quality, A/C units provide an easy way to decrease indoor temperatures, thus A/C is considered an adaptive mechanism that reduces mortality and morbidity [16, 23, 50, 51]. However, A/C use also has negative effects: it increases greenhouse gas emissions [41] and might contribute to “sick building syndrome” and related health issues [52, 53]. Moreover, several literature reviews found limited evidence of A/C’s protective effects [6, 41]. The reason might be that A/C ownership is only the first step, with only wealthier people able to take the second step and keep an A/C on throughout the heatwaves [23, 54–56]. Overall, the literature suggests that A/C ownership might not translate into A/C usage.

### Health and wellbeing

Many epidemiological studies point to increased heat-related mortality risk or the number of negative heat-related experiences among people with poor health [5, 15, 29, 57, 58]. Health status remains a crucial factor influencing heat’s impact, as health affects the regulation of body core temperature [59]. Moreover, high air temperatures can worsen preexisting health conditions, especially cardiorespiratory, kidney diseases or electrolyte disorders [20]. As socioeconomic inequalities directly translate into health inequalities [60], older adults, particularly those with lower socioeconomic status, are more likely to experience chronic diseases, and chronic diseases are often exacerbated by heat stress [6, 20, 61].

Cardiovascular and respiratory illnesses are often indicated as risk factors for heat-related mortality [5, 15, 62, 63]. In terms of individual perception, people with hypertension are more likely to report experiencing the effects of heat on their health [29, 64]. Long-term diabetes may impair people’s thermoregulation [59] and thus progressively increase their heat-related mortality risk [64, 65]. Mental health illnesses, e.g., depression [58], may also increase heat-related mortality [66]. This might happen due to decreased motivation and cognitive ability to take heat-preventing actions [41] as well as medication use that might exacerbate negative heat stress outcomes [67]. Furthermore, depression often coexists with social isolation [68] and is more likely among people with lower SES [69]. Thus, chronic diseases interact with other risk factors, increasing health risk and potentially also negative heat-related experiences.

Obesity is often associated with cardiovascular problems and diabetes, impairing the body’s ability to thermoregulate [70]. Also, obesity is more prevalent among people of lower socioeconomic status [71]. However, there is no consensus on obesity and heat-related mortality risk e.g., while Vandentorren et al. [72] found obesity to slightly increase risk, Semenza et al. [73] results show high BMI having no significant impact on risk. In terms of experiences, studies show that those with obesity are more likely to report that heat impacts their health [64, 74], suggesting that maybe heat-related mortality risk does not rise with weight, but discomfort does.

Regular physical activity may help protect against negative heat impacts [75], as exercise improves general health status and prevents chronic disease [76]. However, physical activity increases core body temperature, thus exercising in a hot environment challenges the thermoregulatory system [59, 77]. Simultaneously, people who exercise regularly may be more aware of their bodies, noticing and reporting more heat experiences [78]. Hence, physical activity’s impact on heat risk and experiences proves unclear.

### Heat-risk perception

The mortality and morbidity analyses, presented above, inform us about risks which typically raise with age: being a woman, having a difficult socio-economic situation and poor health. We also included studies on experiences and perceptions, which prove important for two reasons: (1) they reflect people’s lived experience of heat; and (2) they may impact people’s actions. Hass, Runkle and Sugg’s [79] scoping review demonstrates that heat risk perception influences both a person’s exposure and behavioural response to excessive heat [see also 80]. Howe et al. [81] showed that a group’s risk perception might be detached from their actual risk e.g., inhabitants of warmer US states and low-income groups perceived their higher heat risks, but older age groups did not. Thus, while we focus on heat risk and heat experiences, we assume that when misaligned they constitute a *vulnerability paradox* that can exacerbate people’s risk.

## Methods

This article stems from an interdisciplinary research project on urban heat in Madrid and Warsaw. We build on and juxtapose three analytical and methodological approaches: (1) epidemiological analysis using daily temperature and mortality data for Warsaw and Madrid; (2) the Ordinary Least Squares (OLS) regression based on the survey conducted in both cities in 2022; and (3) the focus group interviews conducted in Warsaw in 2021. The epidemiological perspective provides relatively objective data on heat-related mortality risk, while the survey and focus groups provide subjective data on older adults’ experiences of heat, and their perceptions of how heat affects their health and wellbeing. While the survey gathered large quota samples in each city, focus group research was conducted only in Warsaw and provides individualized, descriptive data that enables a more in-depth understanding of people’s experiences.

### Epidemiological analysis

The epidemiological analysis investigates the short-term associations between exposure to heat and mortality in Warsaw and Madrid, in terms of relative risk (RR) of heat related mortality. RR represents the increase or decrease in the outcome when average temperatures change from the minimum mortality temperature (MMT) to the 90th (moderate heat) and 99th (extreme heat) percentiles. Optimal temperature or MMT refers to the value of mean temperature where temperature-associated mortality is at a minimum. This value varies between different cities. We determine the MMT empirically, based on the fitted model. Daily mean air temperature values were extracted from the HadISD.3.3.0 sub-daily, network of in-situ observations dataset, developed by the UK Meteorological Office– Hadley Center [82]. We use point-data from the meteorological stations that are closest to the two cities. This approach assumes the same daily temperatures across the entire city. Daily all-cause mortality data was collected in Madrid from 01.01.2010 to 31.12.2019 (106420 total deaths May-September), and in Warsaw from 01.01.2002 to 31.12.2018 (121240 total deaths May-September). The data was stratified by age and sex and only data from warm months (May through September) were used in the analysis.

Since we are solely interested in short-term heat-related mortality, we define the lag window as 0–3 days. We estimate the exposure-lag-response associations, using quasi-Poisson regression and distributed lag non-linear models (DLNMs). DLNMs provide a robust and flexible technique to estimate complex non-linear and lagged dependencies [83]. We fit the model for men and women in three age groups (25–64, 65–84 and above 85 years old) for each city, separately. We model the exposure-response curve using a natural spline function with two internal knots at the 50th and 90th percentile of the two cities’ area-specific distributions. We control for seasonality and long-term trends using an interaction term between a natural spline of an indicator for the day of the year (with four degrees of freedom) and a year indicator (as a factor). The minimum mortality temperature (MMT), also referred to as the optimal temperature, corresponds to a minimum mortality percentile between the first and the 99th percentiles. We derived MMT from the predicted overall cumulative exposure-response association in each city and used it as a reference for computing the heat related mortality risk. This modelling approach is inspired by the work of Gasparrini et al. [84].

### Survey

The data comes from “A thermosurvey of older adults’ experiences, perspectives and adaptation to urban heat and climate change”, which was carried out in Warsaw (Poland) and Madrid (Spain) in July-September 2022 [85, 86]. The survey used the Computer Assisted Personal Interviews (CAPI) method (interviewers filled out an online form during a face-to-face interview) on a quota sample by sex and 5-year age groups (65–69; 70–74; 75–79; 80–84; 85+); each quota group included around 100 interviews in each city. The study collected 1,050 interviews in Warsaw and 1,061 interviews in Madrid. The statistics we present use data weighted against age and sex according to Census 2021 results.

We used OLS regression to identify factors influencing how people experience heat, based on their own reporting. Our dependent variable is the number (taking the value from 0 to 11) of heat-related experiences, or more precisely “Yes” answers to the question: *During increased heat*,* do you experience [… e.g. headaches] more often or more strongly than usual?* We assume that the more heat-related experiences respondents have, the more heat stressed they are. We include 11 heat-related experiences: (1) sweating or clammy skin; (2) dry, red skin or a rash; (3) muscle cramps; (4) headache; (5) dizziness, sudden blurred vision; (6) nausea or vomiting; (7) weakness or fatigue; (8) confusion; (9) palpitations or rapid heartbeat; (10) chest pain; 11) shortness of breath or difficulty breathing. These are experiences identified as possible symptoms of a heat-related illness such as heat exhaustion or heat stroke [29, 59, 87].

For explanatory variables, we used individual demographic characteristics (sex, age, marital status, structure of household), socioeconomic status (education level, financial situation, A/C use), health and wellbeing factors (self-rated health status, cardiovascular problems, hypertension, diabetes, respiratory problems, depression, BMI, smoking, physical activity). We focus on A/C using instead of A/C having because many older adults are reluctant to use A/C even if they have it installed (e.g., due to electricity prices), and we are interested in it as both an adaptive strategy and as a socioeconomic status indicator. Summary statistics on all the variables can be found in Appendices A-C. As a robustness check we tested for collinearity and obtained satisfactory values of the variance inflation factor (VIF).

### Focus groups

The focus groups were conducted only in Warsaw, and not Madrid, due to a different project design for each city. The 14 focus groups were conducted in July and August 2021 with 81 older adults living in Warsaw. Participants were diversified according to age (24 people aged 65–69; 24 aged 70–79, and 33 aged 80+); gender (59 women and 22 men); SES (based on a self-declared five-point scale describing their expenses and financial comfort); area of residence. The group interviews centered on people’s perceptions and experiences of urban heat; their bodily, mental and physical reactions, as well as their adaptation strategies. This data is qualitative and therefore aims to provide in-depth findings about people’s opinions, knowledge and experiences. Conducting group interviews, rather than individual ones, aims to foster discussion between the participants and to catch the main, repetitive themes and the nuances between people’s experiences. The conversations were recorded, transcribed verbatim and later analyzed, through the combination of inductive and deductive approaches. We translated the quotes from Polish to English. We use pseudonyms to protect the anonymity of research participants.

## Results

We organized the Results into three interrelated sections. We start by focusing on (1) the role of demographic factors such as sex/gender^1^ and age in how people experience heat as well as how they modify the mortality risks. Then we analyze the impact of (2) socioeconomic factors such as income, education or A/C usage and (3) people’s self-reported health and wellbeing.

### Sex/gender, age and isolation

**Fig. 1:**
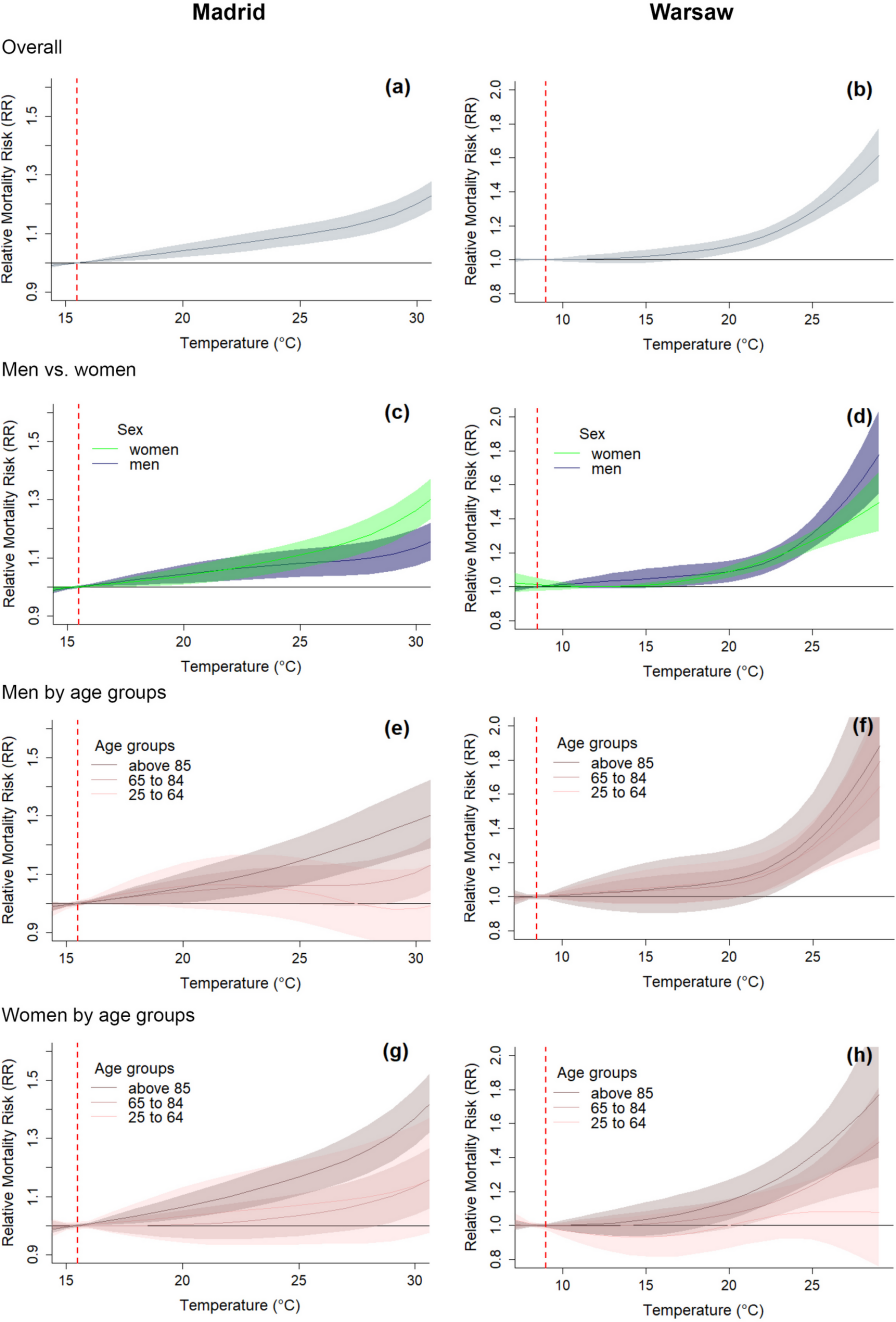
Exposure-response associations: Overall cumulative associations for Madrid (**a**) and Warsaw (**b**); estimation of heat related mortality risk by sex in Madrid (**c**) and Warsaw (**d**); estimation of mortality risk by age group for men in Madrid (**e**) and Warsaw (**f**) and for women in Madrid (**g**) and Warsaw (**h**). The dashed red line marks the MMT temperature

The epidemiological analysis shows an increase in relative mortality risk (RR) with temperature and how age and sex are influencing the patterns of heat related mortality in Warsaw and Madrid. The epidemiological association between mortality and temperature across all demographic groups indicates the overall MMT, based on the estimated exposure-response curves at 15.5^°^C (approximately 5th percentile) in Madrid and at 8.5^°^C (approximately 2nd percentile) in Warsaw. In Warsaw, the overall cumulative heat-mortality association was 1.16 [95% CI: 1.11–1.21] for moderate heat (at the 90th percentile– 22.7^°^C) and 1.39 [95% CI: 1.31–1.48] for extreme heat (at the 99th percentile– 26.5^°^C). These are marginally lower than the estimates for Madrid, where the 90th percentile (30.7^°^C) RR for the whole population is estimated at 1.23 [95% CI: 1.18–1.28] and the 99th percentile (33.6^°^C) RR at 1.44 [95% CI: 1.36–1.53] (Fig. 1). The RR values and their confidence intervals in both cities are greater than 1, with 99th percentile values being higher than the 90th percentile, indicating a robust increase in risk with rising temperatures.

The exposure-response associations suggest more pronounced differences across age and sex groups in Madrid. This could be due to larger age and sex inequalities in Madrid compared to Warsaw.

Our results show heat related mortality risk increasing with temperature in both cities. Panels (a) and (b) in Fig. 1 display the overall exposure-response relationships between daily mean temperature and all-cause mortality in Madrid (a) and Warsaw (b), showing a clear upward trend in relative mortality risk (RR) with increasing temperature. The MMT values (15.5 °C in Madrid and 8.5 °C in Warsaw), indicated by the dashed red lines in all panels of Fig. 1, were estimated empirically from these overall exposure-response curves and used as reference points in the subsequent analyses of mortality risk stratified by sex and age groups. The exposure-response curves, stratified by sex show a robust positive association at temperatures above the MMT for men and women in Madrid (Fig. 1c) and Warsaw (Fig. 1d). While women seemed to be more vulnerable to heat than men in Madrid, a contrasting pattern was found in Warsaw. When it comes to age, we observe a pattern consistent with literature, that shows mortality risk increasing with age for both men and women. In Madrid, women aged 85 + show the highest risks estimates, with RR of 1.42 [95% CI: 1.32–1.53] at the 90th percentile and 1.76 [95% CI: 1.59–1.95] at the 99th percentile (Fig. 1g). The corresponding risks for men aged 85 + tend to be lower, with RR of 1.30 [95% CI: 1.19–1.43] at the 90th percentile and 1.39 [95% CI: 1.21–1.59] at the 99th percentile. Among populations aged 65 to 84, the relationship with temperature became robust only above 27 °C, while for population groups aged 25 to 64, the RR estimates in Madrid were not robust across the observed temperature range.

In Warsaw, while the overall heat–mortality associations remain evident, vulnerability patterns across age groups within each sex appear to be more similar compared to Madrid (Fig. 1f and h). The stratified curves suggest smaller and less consistent differences in risk between age groups for both men and women. Figure 1f presents exposure-response curves for men revealing similar trends across all three age groups. Mortality risks for younger men (25–64) prove similar to mortality risks for older men (65–84 and 85+). The RR for men aged 25 to 64 at the 90th percentile is robust and at 1.16 [95% CI: 1.05–1.29], at the 99th percentile raises to 1.40 [95% CI: 1.20–1.63]. By comparison, the RR for men aged 65 to 84 at the 90th percentile is 1.15 [95% CI: 1.06–1.24], at the 99th percentile it raises to 1.45 [95% CI: 1.29–1.64]. Finally, the RR for men aged 85 + at the 90th percentile is 1.19 [95% CI: 1.19–1.38], at the 99th percentile it raises to 1.52 [95% CI: 1.22–1.89]. In contrast, the patterns shown by the exposure-response curves for women in Warsaw (Fig. 1h) follow more closely the anticipated patterns: the younger age group (25 to 64 years) exhibits the lowest RR (RR 90p: 1.13 [95% CI: 1.03–1.24] and RR 99p: 1.16 [95% CI: 0.98–1.36]), and the oldest age group (above 85 years) demonstrates the highest RR (RR 90p: 1.14 [95% CI: 1.08–1.22] and RR 99p: 1.53 [95% CI: 1.33–1.76]). Thus, while the general trend seems to be in line with literature, with the age group above 85 years being most at risk, this trend is much more evident in the results from Madrid.

In contrast to the epidemiological analysis of heat-related mortality the survey results (Table 1) indicate that women not only in Madrid but also in Warsaw report more types of heat-related experiences than men (around 0,6 more on average, *p* < 0,01). In the case of age, the OLS regression estimates that older respondents reported fewer heat-related experiences, but the relationship is negligible for Warsaw and significant– yet still small– in Madrid (*p* < 0,05). Thus, in Madrid, it seems that those who are at higher risk according to epidemiological analysis, feel slightly fewer heat-related experiences that could alert them of the danger.

**Table 1:** Factors associated with reporting more types of heat-related experiences among older adults living in Warsaw and Madrid (OLS regression)

Variables	Warsaw			Madrid	
	coef.	RSE		coef.	RSE
**Demographic factors**					
Female (vs. Male)	0.677***	(0.161)		0.653***	(0.156)
Older age	-0.00310	(0.0111)		-0.0226**	(0.0111)
Married or in a partnership (vs. not)	0.0782	(0.156)		-0.271*	(0.158)
Lives alone (vs. lives with someone)	-0.457***	(0.173)		-0.366*	(0.199)
**Socioeconomic situation**					
Secondary education (vs. up to primary)	-3.120***	(1.166)		0.569***	(0.216)
Tertiary or postsecondary education (vs. up to primary)	-2.823**	(1.173)		0.531**	(0.219)
Good financial situation (vs. Medium or worse financial situation^a^)	-0.732***	(0.222)		-0.506***	(0.192)
Very good financial situation (vs. Medium or worse financial situation^a^)	-0.715***	(0.249)		-0.893***	(0.215)
Uses air conditioning during heat (almost) always (vs. using it less often which includes never due to not having air conditioning)	0.891***	(0.237)		0.697***	(0.158)
**Health and wellbeing**					
Self-rated health on a 0–10 scale^b^	-0.296***	(0.0615)		-0.226***	(0.0537)
Reports cardiovascular problems	0.564***	(0.204)		0.978***	(0.227)
Reports high blood pressure or hypertension	0.113	(0.183)		-0.121	(0.163)
Reports diabetes or high blood sugar	0.298	(0.216)		0.370**	(0.163)
Reports respiratory problems	1.114***	(0.273)		0.114	(0.231)
Reports depression	1.801***	(0.387)		0.718***	(0.241)
Having obesity according to BMI (vs. Non-obese)	0.239	(0.287)		0.0174	(0.325)
Smokes regularly (vs. does not smoke regularly)	-0.166	(0.284)		0.206	(0.221)
Physically active between once a week or more often (vs. less than once a week)	0.523***	(0.171)		0.597***	(0.174)
Observations	677			819	
R-squared	0.315			0.186	
Prob > F	0			0	
*** *p* < 0.01, ** *p* < 0.05, * *p* < 0.1; RSE = Robust standard errors					

^a^ Financial situation as determined by the answer to: How would you rate the overall financial situation of your household? Very good financial situation: “There is enough for everything without saving in any particular way”; Good financial situation: “I live (we live) frugally and there is enough for everything.; Reference answers: “I live (we live) very frugally to save for bigger purchases.“; “There is enough money only for the cheapest food and clothes”; “There is enough money only for the cheapest food, not enough for clothes.“; “There is not enough money even for the cheapest food or clothes.”

^b^ When evaluating self-rated health, the respondents were told that 0 is the worst imaginable health status and 10 is the best imaginable health status

Source: Own calculations based on data from “A thermosurvey of older adults’ experiences, perspectives and adaptation to urban heat and climate change” [86], data weighted by age and sex for representativity of the city’s older adults

Focus groups provide two interesting age-related findings. First, almost all participants indicated that age affects their experiences of heat. They spoke of physical tiredness, mental fatigue, sweating, and difficulties with breathing. 71-year-old Barbara mentioned that when she returns from shopping on a hot day, *I put the groceries away*,* I sit down and then I feel very*,* very tired. And I might need to sit for up to two hours to recover*. Participants described experiencing more negative consequences of heat, especially getting tired more easily, compared to when they were younger, and noticed different possible reasons for that. They recognized that the heatwaves are more frequent and intense. But also, they pointed out they are impacted more due to their older age. When asked why age makes such a difference, many mentioned worse health and illnesses. One participant, 72-year-old Henryk, pointed to the physiology changing with age, which is in line with the existing research on thermoregulation [e.g. 27, 59]. He explained:

*It all accumulates [in a body]. Everyone at our age has smaller or larger afflictions of various types that burden our bodies*,* and during such extreme temperatures as now*,* such conditions intensify the negative effects on our health. You cannot really speak of any comfort. Younger people do not have as many ailments and they can handle it [the heat] much better*,* that’s obvious.*

The participants were in general aware that age makes them more vulnerable to heat and they experienced it.

*Wanda: Age matters*,* it does its thing*,* and I will stand by it that age impacts* [the experience of heat] *a lot* [to which others nodded and agreed]. *Now* [with age] *a person has more illnesses*,* which unfortunately affect us.*

*Maria: I am sorry*,* but that is not exactly true*,* it is very individualized.*

According to research participants, the effect of age is indirect, as it works through illnesses that one acquires when getting older, and thus it is highly individual. The second age-related finding demonstrates that the oldest group, 80+, spoke about their negative heat experiences less than the youngest group (65–69). Maybe they did not want to complain, or they were already adapted and used to being older. Or maybe only the most resilient individuals reach the age of 80, making them an exceptional group.

Regarding gender, both men and women in group interviews discussed how heat affects their bodies and daily life. They mentioned feeling sleepy, tired and heavy. Many talked about problems with breathing and feeling suffocated when facing urban heat. In terms of bodily experiences, women talked more about swollen legs, headaches and generally feeling tired and sweaty. Men mentioned feeling tired and the discomfort of sweat, although as the transcripts show, they were less outspoken about that than women, which might be more influenced by the cultural stereotypes and expectations about gender, not necessarily showing that they experience less bodily discomfort than women.

Heat affects women’s routines to a larger extent than those of men, as they do most of the invisible, unpaid labor, including housework and care work towards family members or neighbors [32]. During focus groups, men described how they adjust the times when they go outside for shopping or exercise, but if they still worked, they could not adjust the hours of work. But when men were asked about home chores the most common answer was *that’s the wife’s work*. Women in focus groups were affected by heat in two ways due to their unpaid domestic labour: more indoor heat exposure and more schedule disruption.

First, while doing housework, for instance, cleaning, cooking or ironing, they were at higher risk of heat stress. As 66-year-old Monika noticed:

*I also wanted to tell you what we shouldn’t do*,* at least I don’t do it in hot weather anymore. I don’t do household chores like cleaning windows*,* ironing… (…) You simply don’t have the strength to do all of that physically… (…) Well*,* you need to be healthy to do that.*

Cooking and baking were mentioned as especially difficult during hot weather as it heated one’s apartment and put additional strain on the body. Though 69-year-old Jolanta noticed:

*Technology is helping us here because now there are ovens that do not emit heat. So you can bake or roast and they don’t give off heat. I don’t have such a thing*,* so I also change my diet during hot weather and prepare completely different dishes so that I don’t have to use the oven.*

Participants discussed changing their diet during hot weather, partly due to limited appetite, and partly to reduce house chores. Still, some cooking and other housework had to be done even during heat.

Second, heat causes women to change their daily housework routines, for instance, they start very early in the morning or late at night. 65-year-old Felicja, as well as other women we talked to, gets up earlier to prepare food before it gets very hot in her kitchen:

*I watch the weather [programs] so that when it’s hot I plan it this way: I marinate some meat overnight*,* I get up in the morning*,* for example at 6 am*,* I turn on the stove and I bake some things. (…) my grandson really likes the roasted bacon I make. I didn’t have time this morning*,* but tomorrow morning I’ll get up at 6 am to prepare it*,* the bacon is already marinating*,* so he can eat it when he comes to see me.*

Social contacts are an important part of people’s lives, and we expect companionship to help in dealing with heat. However, survey data show a different result. In Madrid both living alone and being in a marriage or partnership slightly decreased the number of heat-related experiences reported (*p* < 0.1). In Warsaw being married/in a partnership had a negligible impact but living alone noticeably decreased the number of heat experiences reported (*p* < 0.01). Focus group interviews contain some possible explanations. As 71-year-old Tadeusz told us: *I only live with my wife […] and when it’s hot and I want to turn on the fan*,* then [I hear] ‘why do you turn on the fan? It’s already enough’. She wears a housecoat*,* and I wear only underwear and it’s hot for me*,* my skin burns.* On the one hand, living together might mean negotiating and compromising around different thermal preferences and cooling strategies, sometimes leaving all sides in some discomfort. On the other hand, other household members sometimes reminded our participants to drink water or use other adaptation strategies, that alone they might have forgone. Similarly, participants mentioned how the other person’s comment can make them more aware of their own heat experiences. Overall, living alone might mean one can adjust the surroundings to fit their preferences, but might also mean a lack of someone to draw their attention to how they feel. Thus, more independence, but maybe less awareness of one’s experiences.

### Socioeconomic situation

We used epidemiological analysis to check how the level of education relates to mortality, but such data were available only for Warsaw, and results show no robust effect of educational attainment (while stratified by age and sex, Appendix D). The survey, however, found statistically significant but contradictory findings. First, the answers of those with primary or lower levels of education differed from others. In Warsaw, respondents with low levels of education reported around three types of heat-related experiences more than the other groups, while in Madrid, respondents with up-to-primary education reported around half of a heat-related experience less than those with secondary or higher education (Table 1). Focus group data suggest no explanations, as education was not asked about nor mentioned during the discussions. Overall, focus groups and epidemiological analysis indicate little importance of education or at least offer no explanations, while the survey indicates contradictory findings in our two case study cities.

In contrast, our results on the importance of the financial situation are consistent. In the survey, a good financial situation (subjectively assessed) translated into fewer heat-related experiences in both cities. In Warsaw, when respondents’ financial standing located above medium, the model predicted they would experience around 0,7 fewer heat-related impacts (*p* < 0,01, Table 1). In Madrid, the model showed a bit more progression, with people in good financial situation predicted to have around 0,5 fewer experiences and those in very good situation around 0,9 (*p* < 0,01). The overall relationship aligns with research carried out at the individual level on heat-related illness [88]. The results from group interviews also confirm this conclusion, perhaps best summed up by 71-year-old Grzegorz:.*there is no escape from the heat*,* there is simply no escape. Well*,* unless someone has loads of money.*.

Our qualitative results pointed to several pathways through which poor financial situation might translate into heat vulnerability. One of them was paid employment. Although the focus groups’ participants were of retirement age, some of the men were still working. Those who did not work mentioned that their retirement status allowed them to organize routines according to their needs and the weather, while those who remained employed had to adjust to their work hours and commute even during the heat. People working inside could sometimes cool their office space. Those working outside, for instance as security guards, could not, placing them in a difficult situation, particularly if they had strict working hours and imposed clothing. 74-year-old Henryk for instance explained:

*From time to time*,* I have such a temporary job where it is especially difficult. Two years ago*,* I remember*,* I had to wear winter clothes in the full August sun. I had to stay for several hours without being able to hide or change anything. It was a nightmare. And it doesn’t even have to be 32 °C. Besides*,* the thermometer is one thing*,* and the sun exposure is another thing….*

Overall, a good financial situation gives people adaptation possibilities, in terms of whether, how and when to work, and where to spend the hottest times.

A/C use is another pathway, through which one’s financial situation can impact heat experience. Unsurprisingly, our survey shows that older adults in Madrid are almost three times more likely to declare using A/C than those in Warsaw (Appendix C), suggesting its role as an adaptive measure. However, the survey-based OLS regression shows that A/C use increased the number of heat-related negative experiences reported in both cities (by 0,891 in Warsaw and 0,697 in Madrid, *p* < 0.01, Table 1). Focus groups provide little guidance as none of the participants owned an A/C. Nevertheless, they did experience it in shops, public transport, friends’ houses and work. Interestingly, while the participants considered A/C bad for one’s health, by causing colds and a harboring bacteria, as well as being noisy, and expensive to use, they did approve of A/C in public spaces, including transport. While many complain about the public A/C temperature being set too low, they preferred it because of the short exposure compared to time spent at home and because of the possibility of changing the means of transportation for a more fitting one. There is a comfort trade-off here between private and public A/C use, similarly to living together or alone case. Public A*/*C is not adapted to the comfort of an individual (e.g. “too low”) but it spares people the direct maintenance efforts and costs. The A/C case shows how adaptations to heat can be organized in different ways impacted by inequality and further impacting both inequality and vulnerability.

### Health status and wellbeing

The survey confirms that in both cities, the better one’s self-rated health (on a 0 to 10 scale) the fewer negative heat-related experiences one reported, revealing health status as an important determinant of heat experience. Our focus group participants often talked about health impacting how they feel the heat, for example as 71-years old Zbigniew explains:

*With age*,* a person becomes ill with something*,* there is no old person to be healthy*,* you can notice that there are […] no young people in queues to see a doctor*,* unless it’s for their grandmother. There are none*,* because they are healthy and do not feel the heat or weather changes. After all*,* when I was a young boy*,* such weather was nothing to me.*

The self-rated health and the general health impact add to the impact of particular conditions that the survey respondents declared having. Out of six preexisting health conditions included in the model (obesity, depression, respiratory problems, diabetes, hypertension and cardiovascular diseases), only two showed a significant impact on the number of heat-related experiences in both cities: depression and cardiovascular problems. Cardiovascular problems increased their predicted number of negative heat experiences by around 0,56 in Warsaw and by almost 1 in Madrid (*p* < 0,01, Table 1), and depression corresponded to an increase in the expected number of heat-related experiences by 1,8 in Warsaw and 0,7 in Madrid (*p* < 0,01). Interestingly, respiratory problems proved significant only in Warsaw, increasing the prediction by 1,1 (*p* < 0,01); while diabetes or high blood sugar was significant only in Madrid leading to an increase of 0,37 (*p* < 0,05). Obesity and high blood pressure had a negligible impact in both cities. Overall, only some preexisting conditions mattered, particularly depression and cardiovascular problems led to more heat-related experiences being declared.

The focus group interviews highlight how important, but also nuanced, is the impact of health on one’s experience. 86-year-old Hanna described how people feel during increased heat:

*I think it depends. Each of us has some ailments. One has rheumatism*,* the other has cardiovascular problems*,* and another person has something else*,* right? And it depends on what you have*,* that’s how the heat feels*,* and that’s where it strikes. It is very much related to it. Because someone could say ‘It’s heat*,* so why do your bones hurt?’ Because it more commonly hurts when it’s cold. But my bones hurt when it’s hot*,* too.*

Similarly, 74-year-old Barbara mentioned: *For me*,* heat is something very noticeable. In the same way as my heart and respiratory problems. I also have Hashimoto’s disease. And because of all those health issues*,* the heat is very tiring for me*. Others indicated that they make conscious decisions to limit heat exposure because of how it affects their health. 65-year-old Jan noticed: *You must adjust to avoid the high temperature. I’m a bit more careful now. I suffer from high blood pressure*,* so I am more careful about when to go out during heat for instance*. While health issues might impact negatively how older adults experience heat, in a few instances hot weather was considered by our research participants as good for one’s health. For instance, 71-year-old Wanda explained, *I have rheumatic problems. I have arthritis. So*,* when it’s hot*,* it’s actually good for me. It’s not very good for my heart*,* but it’s good for my arthritis*. Thus, both the survey data and even more focus group data, point towards an important yet complex impact of different health issues on one’s heat experience.

Returning to the survey outcomes (shown in Table 1), we found two results surprising. First, smoking seemed to have no impact on older adults’ declared heat experiences. Second, people who were physically active every week were also more likely to report more types of heat experiences. In both cities, the model predicted around 0,5 more negative heat experiences for active people in comparison to those who exercised less than once a week (*p* < 0.01). While physical activity was discussed during focus group interviews, it provides little explanation for this result. However, we can hypothesise that, people who are physically active might spend more time outdoors, as in focus groups people talked mainly about gardening, walking, cycling, and engaging in physical activities outside. However, many participants mentioned changing their sports routines due to heat, for instance going out walking or cycling early in the morning or late in the evening, to avoid heat. The second possible explanation is that physically active people are more reflexive on and tuned to their bodily experiences, and hence report more heat-related experiences when asked.

## Discussion

We analyzed how heat affects older adults (65+) in Warsaw and Madrid, and how inequalities impact their heat vulnerability. By combining three methodological approaches: epidemiological analysis of daily mortality data, survey-based OLS regression and focus group interviews, we reveal differences and similarities between people’s heat-related mortality risk and their experiences of heat. We noticed what we call *vulnerability paradoxes*, instances when certain population groups face high mortality risks but report less heat-related experiences. Such mismatches might aggravate people’s vulnerabilities.

However, let us start by discussing the consistent results. First, a good financial situation decreases both the mortality risk [as shown in the literature by e.g. 6], as well as the number of negative experiences of heat. Similarly good health helped both in terms of objective risk and subjective heat experiences, while cardiovascular problems as well as depression were potential risk factors that showed also in more negative heat experiences. Both these results highlight the value of reducing financial and health inequalities as a pathway to greater resilience and improved wellbeing.

Other results worth discussing include being physically active and using A/C. Regular exercise should provide better health and heat resilience. However, physically active respondents reported more heat-related negative experiences, which we explain by potentially increased exposure to the outdoors, and by higher body awareness, however still more research is needed. Similarly, since the literature indicates that A/C ownership protects from heat [16, 23, 50], we expected A/C usage to protect from both the objective risks and negative heat experiences. However, survey results show that A/C users typically reported more negative heat-related experiences. Possibly, people who experience and notice more heat impacts might be more likely to invest in A/C usage, making our results the effect of selection bias. Another explanation might be that regular A/C usage prevents people’s bodies from adapting to the summer heat [89], making going outside a thermal shock that increases noticed heat impacts. Moreover, some of the symptoms we asked about in relation to heat might be caused also by the A/C e.g. headaches, problems breathing, and fatigue [53]. Since some studies [e.g. 90] found no long-term reduction in mortality despite higher A/C ownership, it seems to be a topic worth further studying.

In terms of sex/gender, most epidemiological studies find women facing higher mortality from heat than men [6]. Our epidemiological analysis confirms this pattern in Madrid, especially for older women, and in Madrid’s survey data women also reported more heat-related experiences than men. In Warsaw, the survey shows similar results, with women reporting around 0,7 more experiences than men (*p* < 0.01, Table 1). Also, focus group data demonstrated that women were more outspoken about the impact of heat on their bodies and well-being. Women’s narratives suggest that they indeed are at higher risk of heat stress due to their domestic labour, including cooking, cleaning, and ironing. However, our epidemiological analysis shows that in Warsaw it is men who seem to face higher mortality risks at temperatures above 25 °C, independent of age, although these results are not robust. Thus gender related results on risk and perception are consistent for Madrid, and ambiguous for Warsaw, suggesting a need for further research to achieve higher study power (by extending the study period) and draw more definite conclusions.

In terms of vulnerability paradoxes, let us discuss age. The epidemiological and survey results for Warsaw seem consistent, with both revealing an unclear, negligible or no dependency between age and heat vulnerability. Similarly, focus group interviews showed how participants connected their age with increased heat vulnerability, but also noted that groups aged 80 + talked about negative heat experiences less extensively than their younger counterparts. Thus, results for Warsaw were consistently unclear. For Madrid, however, our epidemiological analysis confirms that, in line with the literature [5, 6], older age slightly increased heat-related mortality risk, while reducing the number of heat experiences reported in the survey. Thus, in Warsaw, where age had little impact on mortality, it also had limited impact on perceptions; but in Madrid, where age seemed to be a risk factor, it also decreased the perception of heat’s impact. Overall, our data demonstrates a discrepancy between mortality risk and subjective experience of heat impacts in Madrid, which may make older adults more vulnerable to heat stress. This leads us to the point that the age paradox exists in some places, particularly hotter ones, and might be contributing to the increased mortality.

The second vulnerability paradox concerns living alone. Living alone at an older age is a mortality risk factor, because there is no one to notice the negative impacts of heat and less chance of calling for help [38, 91]. However, in both cities living alone decreased the predicted number of heat-related experiences (in Madrid at *p* < 0.1 and in Warsaw at *p* < 0.01). Thus, people living alone, who are potentially at higher risk, feel or at least report fewer negative heat impacts on their bodies. We interpret this as either a sign of self-selection so that those who are more independent and less susceptible to heat or illness are more likely to live alone. Another possible interpretation is that older adults who live alone are less aware of their heat-related experiences, which might make them more vulnerable.

Despite limited data availability, which did not allow the use of the same factors in all three methods, it was possible to identify discrepancies between factors influencing mortality risk, and those impacting negative heat-related experiences. The identification of the aforementioned vulnerability paradoxes through the lens of different inequalities was only possible by using a mixed methods approach.

## Conclusions

Though in general adults above 65 years old are more at risk of heat-related morbidity and mortality, they are not a homogenous group and there are many differences in what makes some of them more vulnerable than others. We identified two vulnerability paradoxes - that is situations when someone might be more at risk but not recognize or experience the negative impact of heat - that affect the oldest group of adults (80+) and older people living on their own. Revealing these paradoxes was only possible by using an interdisciplinary approach. Such paradoxes should be studied locally and taken into account when developing strategies and adaptation measures to protect vulnerable people from heat-related illnesses and deaths.

## Electronic supplementary material

Below is the link to the electronic supplementary material.

Supplementary Material 1

Supplementary Material 2

Supplementary Material 3

Supplementary Material 4

## Data Availability

The anonymized survey dataset analysed during the current study are available in the Social Data Repository (rds.icm.edu.pl) at 10.18150/JZZ7NR. The data from the focus groups will be available upon reasonable request. The mortality data from Warsaw were provided by the Statistics Poland. Anonymized individual daily records of mortality from Madrid were provided by the National Institute of Statistics in Spain (INE, Instituto Nacional de Estadística) after formal agreement approval, which prevents their public release. These records were then aggregated into daily time series for the analysis.

## Footnotes

1 We refer to biological sex when analyzing the quantitative data and use gender when analyzing the qualitative data, where we are more interested in the social and cultural gender expectations and contexts.

## Abbreviations

CAPI: Computer Assisted Personal Interviews
OLS: Ordinary Least Squares
MMT: minimum mortality temperature
RR: relative mortality risk
SES: socioeconomic status
A/C: air conditioning
Dfb: Warm–summer Humid Continental Climate
Csa: Hot–summer Mediterranean climate
IMiGW: Instytut Meteorologii i Gospodarki Wodnej
AEMET: Agencia Estatal de Meteorologia
