# Supplementary material for: From inequalities to vulnerability paradoxes: juxtaposing older adults’ heat mortality risk and heat experiences
Source: Environ Health. 2025 Apr 26;24:24. doi: 10.1186/s12940-025-01179-2 (PMC12034184; doi:10.1186/s12940-025-01179-2)
Supplement: Supplementary file 1 — Supplementary Material 1 [file 12940_2025_1179_MOESM1_ESM.docx]

Appendix A. List of declared experiences – ordered from most popular.

| Warsaw | | |  | Madrid | | |
| --- | --- | --- | --- | --- | --- | --- |
|  | % | N |  |  | % | N |
| sweating or clammy skin | 61.16 | 1035 |  | sweating or clammy skin | 82.92 | 1035 |
| weakness or fatigue | 54.41 | 1003 |  | weakness or fatigue | 66.93 | 975 |
| headache | 30.78 | 997 |  | headache | 39.21 | 950 |
| shortness of breath or difficulty breathing | 25.99 | 1029 |  | shortness of breath or difficulty breathing | 33.47 | 1012 |
| palpitations or rapid heartbeat | 24.62 | 1019 |  | dry skin | 30.49 | 981 |
| dizziness | 17.07 | 1024 |  | dizziness | 26.06 | 1020 |
| dry skin | 16.06 | 1027 |  | palpitations or rapid heartbeat | 22.61 | 979 |
| muscle cramps | 15.38 | 1002 |  | muscle cramps | 14.1 | 1000 |
| confusion | 11.19 | 1018 |  | chest pain | 13.55 | 975 |
| chest pain | 11.08 | 1019 |  | confusion | 11.39 | 1001 |
| nausea or vomiting | 5.41 | 1025 |  | nausea or vomiting | 6.75 | 992 |

Source: Own calculations based on data from “A thermosurvey of older adults’ experiences, perspectives and adaptation to urban heat and climate change” [86], data weighted by age and sex for representativity of the city’s older adults.
