# Supplementary material for: From inequalities to vulnerability paradoxes: juxtaposing older adults’ heat mortality risk and heat experiences
Source: Environ Health. 2025 Apr 26;24:24. doi: 10.1186/s12940-025-01179-2 (PMC12034184; doi:10.1186/s12940-025-01179-2)
Supplement: Supplementary file 2 — Supplementary Material 2 [file 12940_2025_1179_MOESM2_ESM.docx]

Appendix B. List of variables used in explanatory analysis.

| Variables | Description |
| --- | --- |
| Dependent variables | |
| number of heat-related experiences | number (0-11) of declared symptoms (characteristic of heat exhaustion or heat stroke or cardiac problems) when is hot from among the following symptoms: sweating or clammy skin, dry skin, muscle cramps, headache, dizziness, nausea or vomiting, weakness or fatigue, confusion, palpitations or rapid heartbeat, chest pain, shortness of breath or difficulty breathing |
| Independent variables | |
| sex | 1 - female, 0 - male; |
| older age | age 65 and above (continuous variable with values ranging from 65 to 99) |
| married/in a partnership  living alone | 1 - having spouse or partner; 0-otherwise  1 – living alone; 0 -  otherwise (living with someone) |
| education level | 1 – low education (primary and lower education as reference category), 2 – medium education (secondary education), 3 – high education (tertiary or postsecondary education); |
| financial situation | determined by answer to: “How would you rate the overall financial situation of your household?”;  1 – medium or worse financial situation (reference category), covers 4 answers: “I live (we live) very frugally to save for bigger purchases” or “There is enough money only for the cheapest food and clothes.” or “There is enough money only for the cheapest food, not enough for clothes” or “There is not enough money even for the cheapest food or clothes”;  2 – good financial situation, covers answer: “I live (we live) frugally and there is enough for everything”;  3 – very good financial situation, covers answer: “There is enough for everything without saving [in any particular way]”; |
| air conditioning | 1-using always or almost always air conditioning when is hot; 0-otherwise (using less or not having air conditioning) |
| SRH | self-rated currently health status on a scale 0-10, where: 0 is the worst imaginable health status and 10 is the best imaginable health status; |
| cardiovascular | 1-reporting cardiovascular diseases; 0-otherwise |
| high blood pressure or  hypertension | 1-reporting high blood pressure or hypertension; 0-otherwise |
| diabetes or high blood sugar | 1-reporting diabetes or high blood sugar; 0-otherwise |
| respiratory problems | 1-reporting respiratory problems; 0-otherwise |
| depression | 1-reporting depression; 0-otherwise |
| obesity | 1- having obesity if BMI >=30kg/m^2^; 0-otherwise |
| smoking | 1 – smoking regularly; 0 - otherwise |
| physical activity | 1- physical activity between once a week or more often; 0-physical activity less than once a week |

Source: Own elaborations based on “A thermosurvey of older adults’ experiences, perspectives and adaptation to urban heat and climate change” [86].
