# Supplementary material for: From inequalities to vulnerability paradoxes: juxtaposing older adults’ heat mortality risk and heat experiences
Source: Environ Health. 2025 Apr 26;24:24. doi: 10.1186/s12940-025-01179-2 (PMC12034184; doi:10.1186/s12940-025-01179-2)
Supplement: Supplementary file 3 — Supplementary Material 3 [file 12940_2025_1179_MOESM3_ESM.docx]

Appendix C**.** Summary statistics of variables used in the explanatory analysis.

|  | Warsaw | | Madrid | |
| --- | --- | --- | --- | --- |
| Binary (=1) and category variables | | | | |
| Variable | % [weighted] | Obs. | % [weighted] | Obs. |
| sex - female | 62.44 | 1050 | 61.04 | 1061 |
| married/in a partnership | 56.62 | 998 | 62.86 | 1053 |
| living alone | 30.97 | 1030 | 18.34 | 1035 |
| educational level - low | 2.02 | 1030 | 20.86 | 1038 |
| educational level - medium | 32.56 |  | 45.80 |  |
| educational level - high | 65.43 |  | 33.35 |  |
| financial situation – medium or worse | 23.12 | 1027 | 34.28 | 998 |
| financial situation – good | 49.60 |  | 42.96 |  |
| financial situation – very good | 27.28 |  | 22.75 |  |
| air conditioning | 17.15 | 1050 | 45.66 | 1061 |
| cardiovascular | 24.87 | 1027 | 22.26 | 1053 |
| high blood pressure or hypertension | 44.33 | 1003 | 39.48 | 1044 |
| diabetes or high blood sugar | 18.88 | 954 | 31.57 | 1037 |
| respiratory problems | 16.19 | 994 | 17.59 | 1038 |
| depression | 9.42 | 905 | 17.42 | 997 |
| obesity | 13.33 | 1001 | 8.01 | 991 |
| smoking | 14.26 | 1048 | 14.14 | 1058 |
| physical activity at least once a week | 76.06 | 1031 | 53.15 | 1050 |
| Continuous variables | | | | |
|  | Mean | Obs. | Mean | Obs. |
| number of heat-related experiences | 2.55 | 1050 | 3.18 | 1061 |
| older age | 76.46 | 1050 | 77.12 | 1061 |
| SRH | 6.59 | 1049 | 6.54 | 1052 |
|  |  |  |  |  |

Source: Own calculations based on data from „A thermosurvey of older adults’ experiences, perspectives and adaptation to urban heat & climate change” [86], data weighted by age and sex for representativity of the city’s older adults.
