# Supplementary material for: From inequalities to vulnerability paradoxes: juxtaposing older adults’ heat mortality risk and heat experiences
Source: Environ Health. 2025 Apr 26;24:24. doi: 10.1186/s12940-025-01179-2 (PMC12034184; doi:10.1186/s12940-025-01179-2)
Supplement: Supplementary file 4 — Supplementary Material 4 [file 12940_2025_1179_MOESM4_ESM.docx]

Appendix D. Relative Mortality Risk (RR) by education and temperature (effect modification) for women and men in Warsaw.
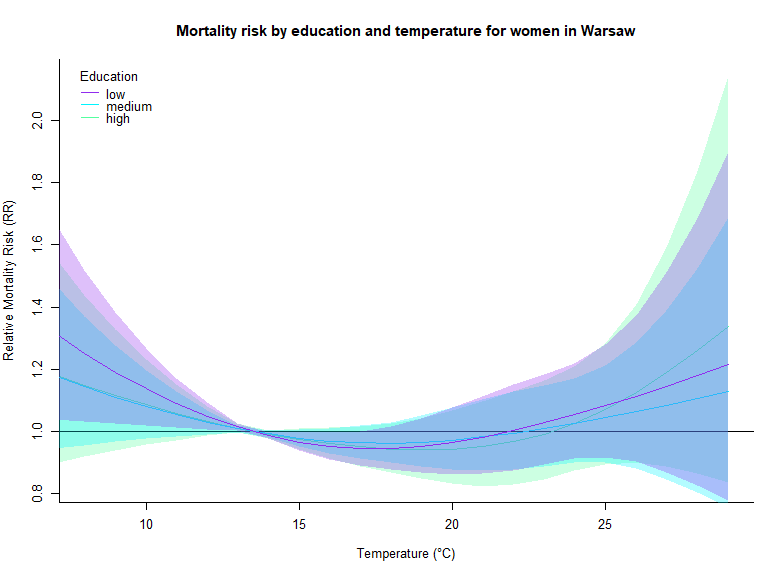

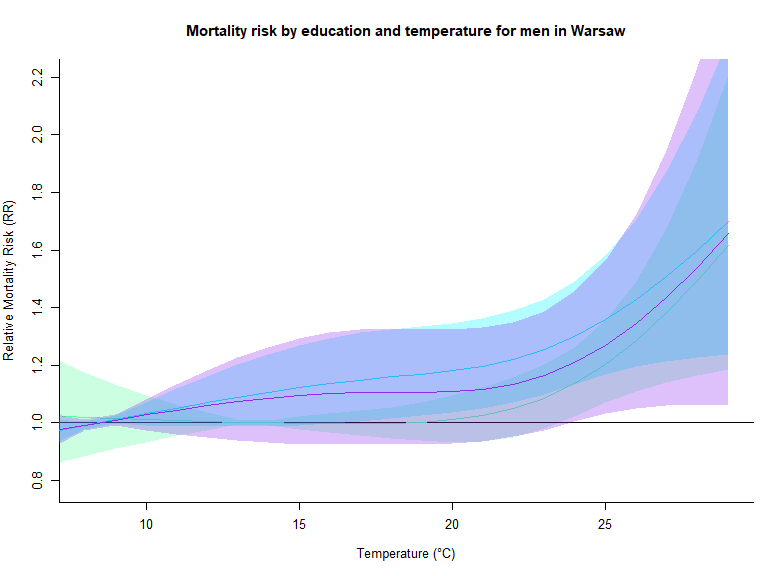


We analyze the effect of education in Warsaw, where the dataset contains mortality by educational attainment, in addition to age and sex. More precisely, the Warsaw dataset is stratified by six categories of educational attainment (6 – incomplete primary, 5 – basic, 4 – vocational, 3 – medium, 2 – post-secondary, 1 – higher and n – no data), which we further aggregated in three categories of low (6 and 5), medium (4 and 3) and high education (2 and 1). The results of this analysis are not robust enough to show a significant effect modification of educational attainment, due to large uncertainty in outcome. Further investigation on more aggregated data, is required to determine the amplitude of the effect modification of education in Warsaw. Such an investigation, however, is beyond the scope of this paper.
